# Real-time estimation of the influenza-associated excess mortality in Hong Kong

**DOI:** 10.1017/S0950268819001067

**Published:** 2019-06-13

**Authors:** Jessica Y. Wong, Edward Goldstein, Vicky J. Fang, Benjamin J. Cowling, Peng Wu

**Affiliations:** 1 WHO Collaborating Centre for Infectious Disease Epidemiology and Control, School of Public Health, Li Ka Shing Faculty of Medicine, The University of Hong Kong, Hong Kong Special Administrative Region, China; 2 Center for Communicable Disease Dynamics, Department of Epidemiology, Harvard TH Chan School of Public Health, Boston, MA, USA

**Keywords:** Death, human influenza, impact

## Abstract

Statistical models are commonly employed in the estimation of influenza-associated excess mortality that, due to various reasons, is often underestimated by laboratory-confirmed influenza deaths reported by healthcare facilities. However, methodology for timely and reliable estimation of that impact remains limited because of the delay in mortality data reporting. We explored real-time estimation of influenza-associated excess mortality by types/subtypes in each year between 2012 and 2018 in Hong Kong using linear regression models fitted to historical mortality and influenza surveillance data. We could predict that during the winter of 2017/2018, there were ~634 (95% confidence interval (CI): (190, 1033)) influenza-associated excess all-cause deaths in Hong Kong in population ⩾18 years, compared to 259 reported laboratory-confirmed deaths. We estimated that influenza was associated with substantial excess deaths in older adults, suggesting the implementation of control measures, such as administration of antivirals and vaccination, in that age group. The approach that we developed appears to provide robust real-time estimates of the impact of influenza circulation and complement surveillance data on laboratory-confirmed deaths. These results improve our understanding of the impact of influenza epidemics and provide a practical approach for a timely estimation of the mortality burden of influenza circulation during an ongoing epidemic.

## Introduction

Influenza virus infections cause a considerable impact on public health. While most infections are mild, a small fraction is severe, resulting in hospitalisation or even death. Worldwide, ~290 000 to 650 000 deaths are attributable to influenza each year [1]. Ecological analyses of mortality rates over time, in combination with surveillance data on influenza activity, are commonly used to estimate influenza-associated mortality [2]. There are large studies on the estimation of influenza-associated excess all-cause mortality, like the EuroMOMO project in Europe since 2008 [3]. However, data on mortality rates are rarely available in near real-time, and this typically prohibits timely estimates of the mortality impact of influenza epidemics.

It has previously been shown that the association between the measure of influenza activity and mortality rates is quite stable across influenza epidemics [4]. Here, we explore the potential for using a combination of a statistical model fitted to historical data as well as real-time information on influenza activity to predict the impact of influenza epidemics in real-time before mortality data become available. We evaluate the model performance using data from Hong Kong from 2006 through 2016.

## Methods

### Sources of data

Age-specific weekly all-cause deaths and the corresponding annual mid-year population estimates between 2006 through 2016 were obtained from the Census and Statistics Department of the Hong Kong Government [5]. Surveillance data on influenza consisted of two data streams: (i) data on influenza-like illness (ILI) from around 50 sentinel private medical practitioners represented by the weekly proportion of outpatients reporting a fever >38 °C plus a cough or sore throat as reported by the Centre for Health Protection (CHP) of the Hong Kong Department of Health, along with (ii) local laboratory data reported by the Public Health Laboratory Services Branch of the CHP on the weekly proportion of specimens from sentinel outpatient clinics and local public hospitals that tested positive for influenza [6]. Surveillance data on influenza deaths were available from the CHP's surveillance systems for paediatric and adult severe cases with laboratory-confirmed influenza virus infection. ILI and laboratory surveillance data stratified by age were not available, whereas severe influenza surveillance data were available by age (<18 years and ⩾18 years).

### Statistical analysis

A linear regression model was used to estimate the influenza-associated excess mortality according to the following regression equation:

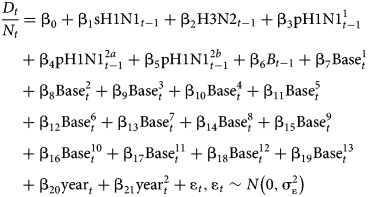

where *t* represents the week number, *D*_*t*_ represents the number of deaths in week *t* and *N*_*t*_ represents the population size in week *t*. *β*_0_ represents the intercept. sH1N1_*t*−1_, H3N2_*t*−1_, 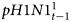, 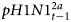, 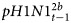 and B_*t*−1_ represent the proxies (covariates) for the weekly incidence of seasonal influenza A(H1N1), influenza A(H3N2), pandemic influenza A(H1N1)pdm09 during the pandemic period in 2009, pandemic influenza A(H1N1)pdm09 in the post-pandemic period and before the 2013–14 influenza season, pandemic influenza A(H1N1)pdm09 on or after the 2013–14 influenza season, and influenza B. These incidence proxies in week *t* are defined as the product of the proportion of respiratory samples that have the given virus detected and the proportion of consultations attributable to ILI from sentinel private medical practitioners in week *t* − 1 respectively since we assumed a time lag of one week between the virus activity and the caused deaths. We split the influenza A(H1N1)pdm09 activity before and after 2013–14 because there was a change in A(H1N1)pdm09 activity before and after 2013–14. 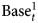, 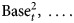 and 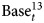 represent the periodic splines with a period of 52 weeks, with a knot every 4 weeks (baseline terms for mortality not attributable to influenza). year_*t*_ and represent the linear and non-linear (quadratic) effect of the calendar year (temporal trend terms in mortality). Errors *ε*_*t*_ were assumed to follow a normal distribution with constant variance over time 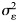.

The influenza-associated excess mortality rates were estimated by subtracting the predicted mortality rate estimated from the fitted regression model setting influenza activity for a type to zero from the predicted mortality rate from the model based on the observed weekly influenza activity. Because the pattern of age-specific proxy measures of influenza activity is generally similar in the different age groups, we included the all-age proxy measure of influenza activity as a covariate in the each regression model. The 95% CIs for excess mortality rates were estimated with a bootstrap approach.

Data on all-cause deaths are not available in real-time in many places, including Hong Kong and Europe [3, 7]. Specifically, we fit a linear model using mortality data and influenza surveillance data from year 1 to year *n*. Then we can use the model, namely the estimates of the regression coefficients for the different covariates corresponding to the different influenza (sub)types for years 1 through *n* to estimate (predict) the number of influenza-associated excess mortality in year *n* + 1 using the available influenza surveillance data for year *n* + 1. We then compared the influenza-associated excess mortality rates estimated in this fashion with the laboratory-confirmed deaths from severe influenza surveillance systems. Additionally, we performed retrospective estimation of excess mortality based on 8 years of data, namely the 2009–2016 period. For the real-time estimation of excess mortality, predictions for each of the 2012 through the 2017 seasons were based on data for *n* = 6 preceding years, while prediction in 2018 was based on 5 preceding years since we only have mortality data until 2016. We assessed the performance of our real-time prediction approach by comparing the real-time mortality estimates for each year (2012 through 2016) with the retrospective estimates for the 2009–2016 period. We also considered models with variations of the main model, including those without the term(s) for the calendar year, without splitting the proxy of pandemic influenza A(H1N1), or splitting the proxy of influenza A(H3N2), and then selected the model with the lowest Akaike information criterion score for real-time estimation. We included seasonal influenza A(H1N1) in the model but would not report the influenza-associated excess mortality estimates of the virus in the main results because it only circulated for one year during the study period. All statistical analyses were conducted in R version 3.3.0 (R Foundation for Statistical Computing, Vienna, Austria).

## Results

Using the product of ILI and laboratory data as the proxy of influenza activity, we estimated the excess all-cause mortality associated with each influenza type and subtype in each year between 2009–2016 in Hong Kong under the linear regression model (retrospective estimation, Table 1). Influenza A(H1N1)pdm09 replaced seasonal influenza A(H1N1) when the first wave of H1N1pdm09 began in summer 2009. In 2009, we estimated that the overall excess death rate associated with H1N1pdm09 was 4.6 (95% CI: −1.9, 10.9) per 100 000 population. Annual estimates of excess deaths associated with influenza A(H3N2), with point estimates of the excess mortality ranging from 3.1 to 17.3 per 100 000 population in 2009 through 2016, tended to be greater than those annual estimates associated with other influenza subtypes.

**Table 1.** Type and subtype-specific influenza-associated excess all-cause mortality rates in each year in Hong Kong based on retrospective data analysis, 2009 to 2016

	Excess mortality rate (per 100 000)							
Year	A(H3N2)	(95% CI)	A(H1N1)pdm09	(95% CI)	B	(95% CI)	All influenza	(95% CI)
2009	6.97	(4.74, 9.14)	4.64	(−1.86, 10.90)	1.12	(0.24, 1.94)	7.02	(−3.49, 18.12)
2010	10.80	(7.34, 14.16)	6.00	(2.03, 10.05)	6.23	(1.35, 10.77)	23.03	(15.46, 30.21)
2011	3.05	(2.07, 4.00)	8.11	(2.74, 13.59)	3.62	(0.79, 6.25)	14.78	(8.36, 21.15)
2012	15.09	(10.25, 19.78)	0.39	(0.13, 0.66)	9.00	(1.96, 15.55)	24.48	(16.03, 32.07)
2013	4.47	(3.04, 5.86)	4.23	(1.97, 6.44)	1.02	(0.22, 1.76)	9.72	(6.68, 12.59)
2014	5.20	(3.53, 6.81)	9.50	(4.42, 13.92)	6.64	(1.44, 11.47)	21.33	(14.93, 27.27)
2015	17.26	(11.73, 22.62)	0.83	(0.39, 1.22)	2.98	(0.65, 5.15)	21.07	(15.15, 27.19)
2016	6.32	(4.30, 8.29)	14.04	(6.53, 20.56)	7.42	(1.61, 12.81)	27.78	(19.58, 35.57)

The average annual excess all-cause mortality estimates associated with influenza in all ages between 2009–2016 was 18.7 (95% CI: 13.3, 24.1) per 100 000 population (Table 2). The older adults had the highest excess mortality among all the age groups. Influenza A(H3N2) was associated with the greatest excess mortality rate among all influenza type/subtypes. The average annual excess mortality estimates associated with influenza A(H3N2) increased with age from approximately zero (point estimate −1.2; 95% CI: −3.2, 0.7) per 100 000 population per year in aged 0–4 years to 55.5 (95% CI: 37.2, 73.6) per 100 000 population per year in aged ⩾65 years.

**Table 2.** Average type and subtype-specific annual excess all-cause mortality rates in different age groups in Hong Kong based on retrospective data analysis, 2009 to 2016

	Average excess mortality rate (per 100 000 population per year)											
Virus	0–4 years	(95% CI)	5–14 years	(95% CI)	15–44 years	(95% CI)	45–64 years	(95% CI)	⩾65 years	(95% CI)	All ages	(95% CI)
All influenza	−0.89	(−5.28, 3.31)	−0.14	(−1.31, 1.05)	−0.10	(−1.31, 1.15)	2.20	(−1.69, 6.12)	128.60	(92.80, 162.23)	18.72	(13.33, 24.07)
A(H3N2)	−1.20	(−3.18, 0.72)	−0.02	(−0.55, 0.56)	0.30	(−0.28, 0.86)	0.90	(−1.01, 2.79)	55.52	(37.18, 73.55)	8.66	(5.88, 11.35)
A(H1N1)pdm09	0.26	(−1.67, 2.18)	−0.20	(−0.74, 0.30)	−0.09	(−0.61, 0.50)	1.55	(−0.26, 3.19)	36.67	(18.50, 51.95)	5.99	(3.41, 8.46)
B	0.18	(−2.26, 2.91)	0.07	(−0.58, 0.83)	−0.40	(−1.18, 0.36)	−0.14	(−2.39, 2.32)	40.39	(15.69, 62.47)	4.77	(1.04, 8.24)

**Fig. 1.**
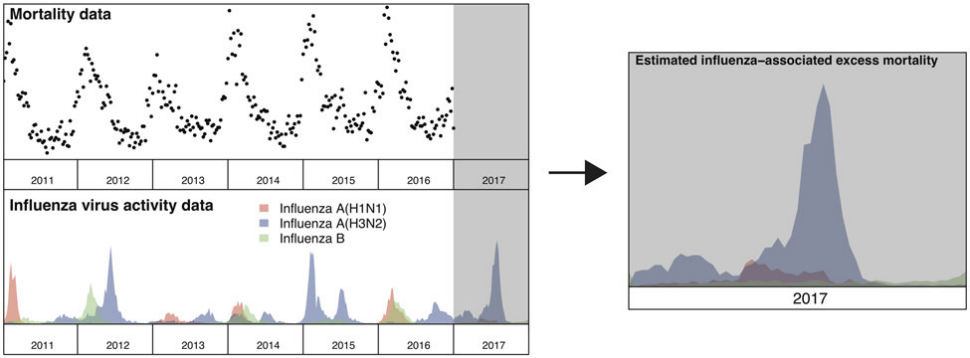
Schematic illustration for real-time prediction of excess mortality in 2017. Step 1: Apply regression model to mortality data (from 2011 to 2016), using influenza virus activity from past years (from 2011 to 2016) as a covariate. Step 2: Predict influenza-associated excess mortality in current year (2017) by applying the fitted model to all year's influenza virus activity data (from 2011 to 2017).

**Fig. 2.**
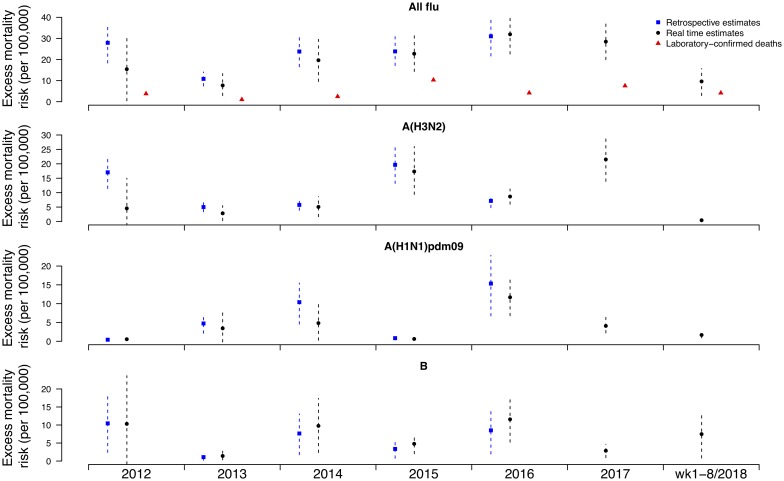
Retrospective and real-time excess all-cause mortality rates *vs.* laboratory-confirmed mortality rates in each year in Hong Kong in population ⩾18 years by virus type and subtype, 2012 to 2018.

**Fig. 3.**
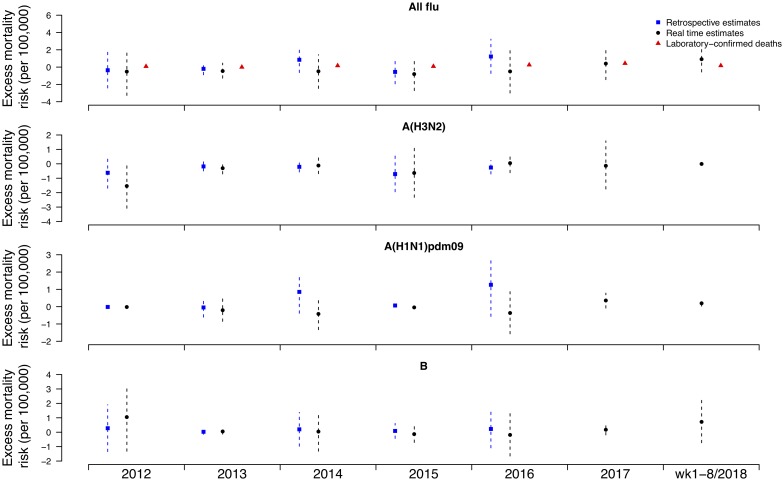
Retrospective and real-time excess all-cause mortality rates *vs*. laboratory-confirmed mortality rates in each year in Hong Kong in population <18 years by virus type and subtype, 2012 to 2018.

Under this new methodology and using 2017 as an example, we estimated influenza-associated mortality in 2017 in real-time based on two components: (i) historical mortality data from 2011 through 2016 and (ii) influenza surveillance data from 2011 through 2017 (Fig. 1). We compared the annual estimates of the excess all-cause influenza-associated mortality rates for the 2009–2016 period (the retrospective estimates), the real-time excess all-cause influenza-associated mortality rates estimated each year and the laboratory-confirmed mortality rates from the severe influenza surveillance system during each year in Hong Kong by virus type and subtype in persons ⩾18 years (Fig. 2; Table 3). Using the real-time approach, we estimated that the overall excess all-cause mortality associated with all influenza was the highest in 2016 for the 2012–2018 period, with the majority of the excess mortality associated with influenza A(H3N2). Although the annual estimates from 2012 through 2018 involve large variations, point estimates of the real-time estimates of excess mortality in each subtype were generally similar to those estimates based on the retrospective approach. CIs of the annual and type/subtype-specific estimates were also similar in both approaches. In comparison, the laboratory-confirmed mortality rates have a similar pattern as the influenza-associated excess mortality rates, with zero to two-fold lower. Among adolescents and young adults, the real-time estimates of excess mortality were comparable with the retrospective estimates of excess mortality, though neither of them generally reached statistical significance (Fig. 3; Table 3). In sensitivity analysis, this approach is also applicable to cause-specific mortality data including respiratory deaths (Fig. S1).

**Table 3.** Retrospective and real-time estimates of influenza-associated excess all-cause mortality rates in Hong Kong in population ⩾18 and <18 years by virus type and subtype, 2012 to 2016

	Excess mortality rate (per 100 000)							
Year	A(H3N2)	(95% CI)	A(H1N1)pdm09	(95% CI)	B	(95% CI)	All flu	(95% CI)
**Retrospective, ⩾18 years**								
2012	17.04	(11.44, 22.43)	0.42	(0.14, 0.69)	10.45	(2.40, 17.87)	27.91	(18.34, 36.54)
2013	5.00	(3.36, 6.58)	4.73	(2.13, 7.17)	1.10	(0.25, 1.87)	10.83	(7.44, 14.09)
2014	5.76	(3.87, 7.58)	10.40	(4.56, 15.48)	7.64	(1.75, 13.07)	23.80	(16.55, 30.62)
2015	19.65	(13.19, 25.86)	0.83	(0.36, 1.24)	3.36	(0.77, 5.75)	23.84	(17.12, 30.83)
2016	7.15	(4.80, 9.40)	15.35	(6.73, 22.85)	8.52	(1.95, 14.56)	31.01	(21.65, 39.84)
**Real-time, ⩾18 years**								
2012	4.56	(−6.09, 15.03)	0.57	(0.22, 0.99)	10.33	(−4.56, 23.69)	15.47	(−2.75, 31.50)
2013	2.84	(0.27, 5.50)	3.45	(−0.19, 7.67)	1.45	(0.32, 2.75)	7.74	(2.88, 13.33)
2014	5.06	(1.67, 8.63)	4.83	(0.26, 9.98)	9.78	(2.38, 17.39)	19.67	(9.58, 29.57)
2015	17.36	(9.37, 26.00)	0.63	(0.34, 0.92)	4.79	(2.03, 7.50)	22.78	(14.32, 32.08)
2016	8.65	(5.98, 11.28)	11.72	(6.78, 16.40)	11.59	(5.24, 17.43)	31.96	(22.58, 40.86)
**Retrospective, <18 years**								
2012	−0.62	(−1.69, 0.55)	−0.02	(−0.08, 0.05)	0.28	(−1.34, 1.91)	−0.36	(−2.42, 1.76)
2013	−0.18	(−0.49, 0.16)	−0.05	(−0.60, 0.53)	0.03	(−0.14, 0.20)	−0.20	(−0.89, 0.59)
2014	−0.21	(−0.57, 0.19)	0.85	(−0.38, 1.91)	0.20	(−0.97, 1.38)	0.85	(−0.63, 2.44)
2015	−0.71	(−1.94, 0.63)	0.07	(−0.03, 0.15)	0.09	(−0.43, 0.61)	−0.55	(−1.94, 0.94)
2016	−0.26	(−0.71, 0.23)	1.26	(−0.57, 2.83)	0.22	(−1.09, 1.54)	1.23	(−0.73, 3.23)
**Real-time, <18 years**								
2012	−1.54	(−3.08, −0.05)	−0.01	(−0.07, 0.05)	1.05	(−1.32, 3.28)	−0.50	(−3.28, 2.10)
2013	−0.30	(−0.70, 0.09)	−0.20	(−0.85, 0.52)	0.06	(−0.13, 0.25)	−0.44	(−1.28, 0.46)
2014	−0.11	(−0.68, 0.47)	−0.41	(−1.33, 0.56)	0.05	(−1.32, 1.45)	−0.48	(−2.47, 1.43)
2015	−0.64	(−2.33, 1.13)	−0.04	(−0.10, 0.04)	−0.13	(−0.71, 0.55)	−0.80	(−2.71, 1.06)
2016	0.06	(−0.62, 0.73)	−0.36	(−1.57, 0.87)	−0.18	(−1.65, 1.32)	−0.49	(−3.00, 1.93)

## Discussion

The influenza-associated all-cause excess mortality rates estimated from this study suggested an annual average of 1340 (95% CI: 954, 1723) excess deaths associated with influenza in Hong Kong from 2009 through 2016, slightly higher than earlier estimates for 1998–2009 [8] and 2004–2006 [9]. The majority of influenza-associated excess deaths occurred in persons aged 65 years or older, comparable to the findings from other countries [2, 10, 11]. Here, we developed methodology for estimating influenza-associated mortality in real-time based on two ingredients: (i) past mortality data and (ii) influenza surveillance data, including real-time surveillance data. The real-time estimates of excess mortality were similar to the retrospective excess mortality estimates, demonstrating the potential of our approach to provide timely information on the impact of influenza circulation on mortality during the course of influenza seasons. In addition, our approach can provide important information to health authorities to improve situation awareness and calibration of public health interventions like vaccination and prescription of antiviral for high-risk individuals, particularly during severe influenza seasons [12, 13]. Because there is usually a delay in obtaining population mortality data, real-time estimation of excess mortality based on historical death data is important for predicting the impact of the influenza viruses circulating in the current season on the population and for planning for public health responses, especially when there is a relatively more intense virus activity and/or an observed substantial impact on the healthcare system [14].

The relation between influenza surveillance data and influenza-associated mortality is measured by the regression coefficients for the different influenza (sub)types in the linear inference model. In a previous study, we showed that the regression coefficients for each influenza type/subtype generally did not change much over time during the study period [4]. In our present study, the implicit assumption when estimating the influenza-associated excess mortality in real time was that the regression coefficients for the major influenza (sub)types are stable. If there is a change in the regression coefficients, possibly due to the change in the strain of the circulating influenza virus, or due to an emerging influenza epidemic, or for other reasons, the application of regression coefficients estimated from past data to the current influenza surveillance data could be questionable, hence the real-time influenza-associated excess mortality could be overestimated or underestimated in our approach. We note that the real-time estimate of influenza A(H3N2)-associated mortality in 2012 is notably lower than the retrospective estimate.

During an evolving influenza epidemic it can be challenging to quantify its impact on mortality and other severe outcomes [15, 16]. Our approach to relate surveillance data to excess mortality provides a way to quantify the mortality burden of the ongoing influenza epidemic. Future work could extend our approach to forecast the mortality burden of the whole epidemic in real-time via forecasting future incidence [17–19]. Providing real-time estimates of the mortality impact of evolving influenza epidemics could help inform public health responses to those epidemics [15, 20].

Our study has a few limitations. First, our proxy measure of influenza activity, obtained by combining ILI data with laboratory surveillance data, constructed as the product of the weekly proportion of outpatient consultations for ILI and the weekly proportion of respiratory specimens testing positive for each influenza by type/subtype, may not accurately measure influenza incidence in the community due to reasons such as changes in healthcare seeking behaviour, or changes in laboratory testing practices over time. In 2009 we found that this proxy measure was closely correlated to hospitalisations for influenza A(H1N1)pdm09 infection [7]. We did not have age-specific surveillance data on ILI or laboratory surveillance data, and the use of all-age surveillance data as the proxy measure of influenza activity as a covariate in each of the regression model might be less optimal for the estimation of the influenza-associated mortality in some age groups. Secondly, the current model only explained 83% of the variation in the all-cause mortality rates, and there is room to further improve the models used in this study. Finally, we did not have information on patterns in influenza vaccination coverage over time, which had increased from low levels to around 10% in 2015 in Hong Kong. Our model may be improved by incorporating the information on vaccination coverage which is likely to vary by age due to various reasons including access to free vaccination, and therefore can be used to investigate the potential impact of vaccination on influenza-associated excess all-cause mortality.

In conclusion, we have described an approach to provide real-time estimates of the mortality impact of influenza epidemics, based on historical mortality data along with historical and real-time information on influenza activity. The performance of this approach could be explored in other locations, and in future this work could be combined with methods for forecasting of influenza incidence to provide forecasts of influenza-associated mortality rates for evolving influenza epidemics and help inform control and mitigation efforts.

## Supplementary material

For supplementary material accompanying this paper visit https://doi.org/10.1017/S0950268819001067. click here to view supplementary material
