# Supplementary figures and images for: Real-time estimation of the influenza-associated excess mortality in Hong Kong
Source: Epidemiol Infect. 2019 Jun 13;147:e217. doi: 10.1017/S0950268819001067 (PMC6627011; doi:10.1017/S0950268819001067)

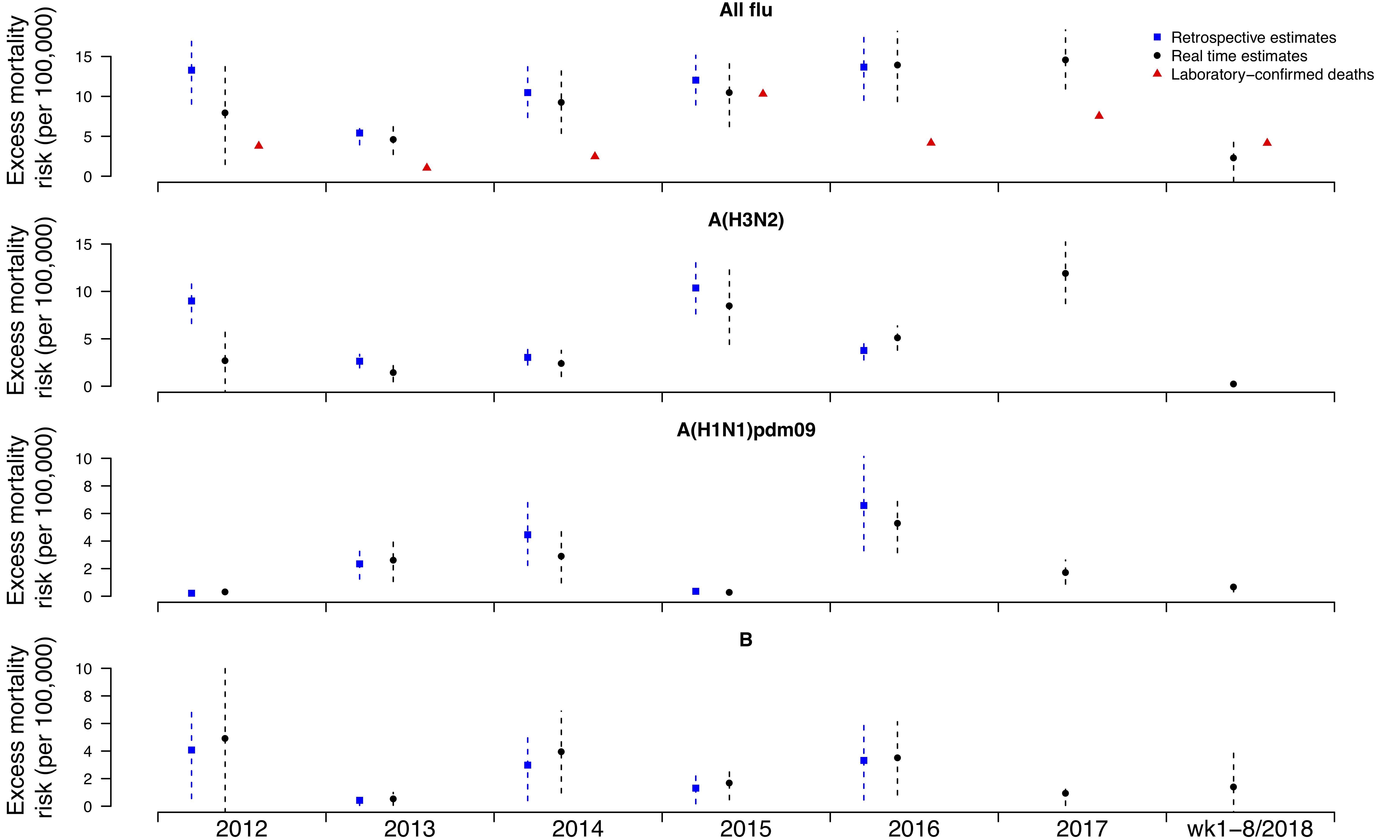

Supplement: Supplementary file 1 [file S0950268819001067sup001.tiff]
